# Physical activity, depressed mood and pregnancy worries in European obese pregnant women: results from the DALI study

**DOI:** 10.1186/s12884-015-0595-z

**Published:** 2015-07-31

**Authors:** Linda de Wit, Judith G. M. Jelsma, Mireille N. M. van Poppel, Annick Bogaerts, David Simmons, Gernot Desoye, Rosa Corcoy, Alexandra Kautzky-Willer, Jürgen Harreiter, Andre van Assche, Roland Devlieger, Dirk Timmerman, David Hill, Peter Damm, Elisabeth R. Mathiesen, Ewa Wender-Ozegowska, Agnieszka Zawiejska, Pablo Rebollo, Annunziata Lapolla, Maria G. Dalfrà, Stefano Del Prato, Alessandra Bertolotto, Fidelma Dunne, Dorte M. Jensen, Liselotte Andersen, Frank J. Snoek

**Affiliations:** Department of Public and Occupational Health, EMGO+ Institute for Health and Care Research, VU University Medical Centre, Van der Boechorststraat 7, 1081BT Amsterdam, The Netherlands; Institute for Sport Science, University of Graz, Graz, Austria; Department of Healthcare Research, PHL University College, Limburg Catholic University College, Hasselt, Belgium; Institute of Metabolic Science, Addenbrookes Hospital, Cambridge, UK; Department of Obstetrics and Gynecology, Medizinische Universität Graz, Graz, Austria; Institut de Recerca de L’Hospital de la Santa Creu i Sant Pau, Barcelona, Spain; CIBER Bioengineering, Biomaterials and Nanotechnology, Instituto de Salud Carlos III, Madrid, Spain; Medical University of Vienna, Vienna City, Austria; KU Leuven Department of Development and Regeneration: Pregnancy, Fetus and Neonate, Gynaecology and Obstetrics, University Hospitals Leuven, Leuven, Belgium; Recherche en Santé Lawson SA, Bronschhofen, Switzerland; University Hospital of Copenhagen - Rigshospitalet, Copenhagen, Denmark; Akademia Medyczna im Karola Marcinkowskiego, Poznan, Poland; BAP Health Outcomes Research SL, Oviedo, Spain; Universita Degli Studi di Padova, Padova, Italy; Università di Pisa, Pisa, Italy; National University of Ireland, Galway, Ireland; Odense University Hospital, Odense, Denmark; Department of Medical Psychology, EMGO+ Institute for Health and Care Research, VU University Medical Centre, Amsterdam, The Netherlands; Department of Medical Psychology, Academic Medical Centre, Amsterdam, The Netherlands

**Keywords:** Exercise, Mental health, Pregnancy, Obesity

## Abstract

**Background:**

The purpose of this study was to examine the association between mental health status (i.e. depressed mood and pregnancy-related worries) and objectively measured physical activity levels in obese pregnant women from seven European countries.

**Methods:**

Baseline data from the vitamin D and lifestyle intervention for the prevention of gestational diabetes mellitus (DALI) study were used. Time spent in moderate-to-vigorous physical activity (MVPA) and sedentary behaviour was measured with accelerometers. Depressed mood was measured with the WHO well-being index (WHO-5) and pregnancy-related worries with the Cambridge Worry Scale (CWS). In addition, socio-demographic characteristics, lifestyle factors, and perceptions and attitude regarding weight management and physical activity were measured. Linear regression analyses were performed to assess the association of mental health status with MVPA and sedentary behaviour.

**Results:**

A total of 98 obese pregnant women from Austria, Belgium, Ireland, Italy, Poland, Spain and the Netherlands were included. Women had a mean age of 31.6 ± 5.8 years, a pre-pregnancy BMI of 34.1 ± 4.3 kg/m^2^, and were on average 15.4 ± 2.8 weeks pregnant. WHO-5 scores indicative of depressed mood (<50) were reported by 27.1 % of the women and most frequently endorsed pregnancy-related worries pertained to own and the baby’s health. Women with good well-being spent 85 % more time in MVPA compared to women with a depressed mood (*P* = 0.03). No differences in MVPA levels were found for women with no, some, or many pregnancy worries. Depressed mood and pregnancy-related worries were not associated with sedentary behaviour.

**Conclusions:**

These findings suggest that in pregnant women who are obese, a depressed mood, but not pregnancy-related worries, may be associated with less physical activity. The combined risk of poor mental health and low physical activity levels makes women vulnerable for pregnancy complications. Whether a depressed mood may be a barrier for improving physical activity warrants further study.

## Background

There is compelling evidence that physical activity during pregnancy is associated with fewer complications during pregnancy and delivery, lower chance of gestational diabetes (GDM), lower rates of hypertension [1–4], and better mental health status [5–8].

The American College of Obstetrics and Gynaecologists (ACOG) recommends pregnant women to be active in moderate intensity activities (3–5 metabolic equivalents) for at least 30 min on most, and preferably all, days of the week [9]. However, adherence to these recommendations appears to be low, with less than 50 % of all women meeting the ACOG recommendations pre-pregnancy and throughout pregnancy in the USA [4, 6, 10] and in the EU [11, 12].

With the growing number of overweight (body mass index (BMI) > 25 kg/m^2^) and obese (BMI > 30 kg/m^2^) women of reproductive age [13], obesity during pregnancy is becoming an increasing problem. Obesity in pregnancy increases the risk of excessive weight gain, and the risk of pregnancy-related complications, such as GDM [14]. In pregnant women who are overweight and obese, physical activity is of even greater importance, since it can help improve these pregnancy outcomes [4, 15, 16].

A better understanding of the factors related to low physical activity levels in pregnant women in general is required in order to maximize acceptability and efficacy of lifestyle interventions [17, 18]. One predictor of low physical activity levels that has been well studied outside of pregnancy is poor emotional well-being signifying depression [19–21]. People with depressive symptoms are likely to be less physically active [19]. Conversely, increasing physical activity can help to reduce depressive symptoms in previously inactive people [20]. Also in pregnancy, research suggests that low levels of physical activity are associated with poorer mental health (reviewed in [18]), but the evidence is less convincing. Studies had either a small sample size [2, 8, 22] or physical activity was measured with a (non-validated) diary or questionnaire [23]. Therefore, in this study we measured physical activity levels objectively in a larger study sample, in order to provide more accurate assessment of the relationship between physical activity and mental health.

In pregnant women who are overweight or obese, a poorer mental health status has been found compared to non-obese women [24, 25]. Feelings of humiliation and medicalization of being obese and pregnant appear to have a negative impact on women’s mental health status [26]. In addition, stigma towards obese individuals may lead to a lower self-esteem and may negatively impact mental health [13, 26, 27]. Pregnancy may be experienced as a distressing period, leading to anxieties. Insecurity about the baby’s health, possibility of miscarriage, and concerns about giving birth are especially reported in the first trimester [28]. Women who are depressed or anxious in pregnancy have a higher risk of adverse pregnancy or birth outcomes [29, 30].

Similar to other populations, obese pregnant women with poor mental health (i.e. depressed mood and pregnancy-related worries) might be less physically active, which may further increase their risk for adverse pregnancy outcomes [18]. Moreover, poor mental health may limit the effectiveness of physical activity interventions in obese pregnant women [31]. Therefore, the aim of this study is to examine the association between mental health status and objectively measured physical activity levels of obese pregnant women. For this purpose, we employed data from the European vitamin D and lifestyle intervention for gestational diabetes mellitus prevention (DALI) study. This allows insight into the association between mental health and physical activity in obese pregnant women throughout Europe.

## Methods

### Participants

For this study, baseline data from the DALI project (ISRCTN70595832) were used. Baseline data were collected between October 31, 2011 and April 30, 2013. The study design and data collection of the DALI project are described in detail elsewhere [32]. In summary, this project aims to identify the best available measures to prevent GDM in an ongoing pregnancy, and therefore a randomised controlled trial was conducted in nine European countries: the United Kingdom, Ireland, the Netherlands, Belgium, Poland, Italy, Spain, Austria, Denmark (2 study centres), with women from a diverse range of socio-economic and ethnic backgrounds. For all nine countries, the study design and procedures were approved by the Medical Ethics committee of the respective centres (Cambridge, United Kingdom: National Research Ethics Service, Norwich Research Ethics Committee; Galway, Ireland: Clinical Research Ethics Committee, Galway University Hospitals; Amsterdam, the Netherlands: Ethical Committee of the VU University Medical Center; Leuven, Belgium: Commissie Medische Ethiek van de Universitaire Ziekenhuizen KU Leuven; Poznan, Poland: Komisja Bioetyczna Przy Uniwersytecie Medycznym im. Karola Marcinkowskiego W Poznaniu; Padua, Italy: Il Comitato Etico per la Sperimentazione Clinica della Provincia di Padova; Barcelona, Spain: Comité Ético de Investigación Clínica de la Fundació de Gestió Sanitaria del Hospital de la Santa Creu i Sant Pau; Vienna, Austria: Ethik Kommission Medizinische Universität Wien;

Copenhagen and Odense, Denmark: De Videnskabsetiske Komiteer D for Region Hovedstaden). Participating hospitals and surrounding midwife, obstetrician and general practices were involved in recruitment. Women were followed for about 7 months (from 10 weeks of pregnancy until delivery). Pregnant women were included if aged 18 years and older, were less than 20 weeks pregnant, had a singleton pregnancy, and had a pre-pregnancy body mass index (BMI) ≥ 29 kg/m^2^ based on self-reported weight and measured height. This BMI criterion was agreed upon following a review of local obesity prevalence to ensure all sites had the potential to recruit sufficient women. In a pilot study, the lifestyle interventions (diet and/or exercise) and measurement procedures were tested in each country. Although women who were diagnosed with GDM at baseline (using International Association of the Diabetes in Pregnancy Study Groups (IADPSG) criteria defined as fasting venous plasma glucose ≥ 5.1 mmol/l and/or 1 h glucose ≥10 mmol/l and/or 2 h glucose ≥ 8.5 mmol/l) [33] were excluded from the DALI intervention study, they were not excluded for analysis presented in this paper.

### Measures

#### Physical activity

Physical activity was objectively measured with an Actigraph GT3X, GT1M or Actitrainer accelerometer (all three devices made by ActiGraph, Pensacola, Florida, USA). Previous validation studies found physical activity estimates from the three devices very comparable [34–36]. Although the GT3X is a tri-axial accelerometer, it was analysed from the vertical plane only to standardize activity estimates across the three accelerometers. All three accelerometers are lightweight devices with a sampling frequency of 60–80 (Hz). In this study we used a 1 min epoch. Accelerations derived from participant’s movements were converted into counts per minute.

Participants were asked to wear the accelerometer from waking up until going to bed, attached to the right hip by an elastic waist belt, for at least 3 days. The Actigraph accelerometer has previously gained validity and reliability in free living conditions in adults for between 3 and 5 days [37]. The participants were asked to remove the accelerometer while swimming, showering or bathing. For non-wearing intervals, participants were instructed to write down in a diary how they spent their time.

Crude data were obtained using ActiLife 6 (ActiGraph, LLC, Pensacola, Florida, USA). Periods with no counts for at least 60 min were defined as non-wear time. Days with less than 480 min activity were labelled as invalid data and excluded for analysis [38]. Only women with data for at least three valid days were included [38]. Using the Freedson cut-off points [39], the number of minutes per day in light (100–1951 counts/min), moderate (1952–5724 counts/min) and vigorous (>5724 counts/min) activity were calculated as well as time spent sedentary (<100 counts/min). These cut-off points are widely used, also in pregnancy [7, 40]. Swimming time, recorded in the diary, was added to MVPA, based on the Ainsworth cut-off points [41], a procedure recommended by Esliger *et al.* [42]. For analysis, the time spent in moderate-to-vigorous activity (MVPA) minutes per day) and in sedentary behaviour (as proportion of total wear time) were used.

#### Mental health

Mental health was assessed based on questionnaire data. Questionnaires were administered in the main language of the site: English (UK and Ireland), Dutch (Netherlands and Belgium), Polish, Italian, Spanish, German (Austria) and Danish.

The WHO well-being index (WHO-5) was used to assess emotional well-being [43]. This uni-dimensional questionnaire contains five statements, describing positive moods (e.g. good spirits, relaxation). Each item was rated on a 6-point Likert scale, ranging from 0 (‘at no time’) to 5 (‘all of the time’) pertaining to the past 2 weeks. Total scores were calculated and standardized, ranging from 0 to 100. A total score of 50 or above was considered as good well-being, while a score below 50 indicated depressed mood. This is not a formal depression diagnosis but signifies likely depression [44]. Cronbach’s alpha was 0.83 for all items. Validated versions of the WHO-5 were available for all the languages required in the study.

Pregnancy-related worries were measured with the Cambridge Worry Scale (CWS) [28]. The CWS has good reliability and validity in pregnant women, and overall a strong association with trait anxiety [28]. This 13-item scale assesses current concerns across four domains: social-medical, socio-economic, health and relationships. Responses were scored on a 6-point Likert-scale, ranging from 0 (‘not a worry) to 5 (‘major worry’) for each item. Total scores (0–70) and domain scores (socio-medical 0–20, socio-economic 0–15, health 0–20 and relationships 0–10) were calculated, with higher scores indicating more worrying. There is no established clinical cut-off point. We stratified total scores into tertiles: no or few worries (score ≤ 13), some worries (score 14–25) and many worries (score ≥ 26). Cronbach’s alpha was 0.86 for all items. Spearman correlation between WHO-5 and total CWS was 0.44. The CWS was only available in English and was translated into all the other languages (without back-translation).

#### Other variables

Socio-demographic information was collected at baseline, including age, country, ethnicity, household composition, education (highest completed level) and work status. In addition, aspects such as parity and lifestyle factors at baseline, (e.g. smoking (yes/no) and alcohol consumption (yes/no)) were assessed.

Pre-pregnancy weight in kilograms divided by height in squared meters (kg/m^2^) was used to calculate Body Mass Index (BMI). Pre-pregnancy weight (kg) was based on self-report, while height was measured at baseline with a stadiometer (SECA 206). Objective height measurement was obtained with an accuracy to the nearest centimeter, and the average value of two measurements was used.

Furthermore, attitude, social support and self-efficacy outcome expectancies (based on the Health Action Process Approach (HAPA) model [45] regarding weight and physical activity management during pregnancy were measured. Women indicated the extent to which they agreed with eight items; each item on a 11-point Likert-scale, ranging from 0 (‘not at all’) to 10 (‘very much’). One attitude item pertained to current weight and one attitude item pertained managing weight. Three items pertained to self-efficacy beliefs regarding weight and physical activity management; one item pertained to social support in physical activity and two items pertained to outcome expectancies regarding weight and physical activity management.

### Statistical analysis

For all variables frequencies, means, and standard deviations were calculated. Median values were presented if a continuous variable was not normally distributed. To compare distribution of variables in the different mental health categories (good emotional well-being/depressed mood and tertiles for pregnancy worries), t-tests and one-way ANOVAs were performed for normally distributed variables, non-parametric tests for not-normally distributed variables, and chi-square tests for dichotomous variables.

To analyze the association between mental health and objective physical activity, linear regression analyses were performed for the WHO-5 well-being index (dichotomised) and the Cambridge Worry Scale (in tertiles) combined in the same model. In addition, associations with the proportion of wear time spent in sedentary behavior were assessed. To test the (possible) clustering effect within countries, multilevel analysis with a random intercept were performed. The country of recruitment and the individuals were the levels in the analyses. Because of the positively-skewed distribution of MVPA (min/day), a natural log transformation was executed for the linear regression analyses. Because of the log transformed dependent variable in the regression analyses, the exponent of the beta minus 1 represents the % of change in MVPA between mental health categories.

Interactions between mental health and age, ethnicity, prepregnancy BMI, smoking, alcohol consumption, and parity were assessed, by adding the interaction terms one by one to the regression model. However, none of the interaction terms was significant (*p* < 0.05).

Factors found to be associated with both mental health and physical activity in previous research were considered potential confounders (age, ethnicity, occupational status, household composition, prepregnancy BMI, smoking, alcohol consumption, gestational age, parity). All the variables were entered in the models one by one. Confounding was defined as a change in the regression coefficient of more than 10 % [46].

After controlling for confounding, the HAPA factors (attitude, self-efficacy, social support and outcome expectancy) were added to the models, in order to assess whether these factors confounded the association between mental health and MVPA and/or sedentary behavior. Significance level of the associations in the final model was set at *P*-value 0.05. The data were analysed using SPSS 20.0 (IBM Corporation, Armonk, NY, USA).

## Results

A total of 258 women were measured at baseline and 132 of them wore an accelerometer (except in the UK and Denmark). Of those who wore accelerometers, 14 had no valid data for 3 days and 20 had software problems. These excluded women (*n* = 34) had a higher pre-pregnancy BMI (mean 40.6 kg/m^2^; *P* < 0.01) and were further along in their pregnancies (16.8 weeks; *P* = 0.03).

Finally, 98 women with valid data were included in the analyses. The mean age was 31.6 ± 5.8 years and most were white/Caucasian (75.5 %) and had at least a secondary education (81.6 %) (Table 1). At baseline measurement, women were 15.4 ± 2.8 weeks pregnant. Just over a quarter (27.1 %) of the total sample (*n* = 26) had a depressed mood (WHO-5 score < 50). These women reported a significantly more positive attitude towards physical activity and scored lower on perceived social support (*P* < 0.05) (Table 1). In addition, women with a depressed mood had more pregnancy-related worries, both overall and for the specific domains (*P* < 0.05) (Table 1).

**Table 1:** Baseline characteristics

Characteristic	Total sample	Depressed mood^a^	Good well-being^a^
	(*n* = 98)	(*n* = 26)	(*n* = 70)
Country (n)			
Spain	20	5	15
Austria	27	5	22
Belgium	16	7	9
Poland	10	4	6
Italy	1	0	1
Ireland	7	1	6
The Netherlands	17	5	12
Age (mean ± SD)	31.6 ± 5.8	30.6 ± 6.8	32.0 ± 5.5
Pre-pregnancy BMI (mean ± SD)	34.1 ± 4.3	35.0 ± 5.7	33.8 ± 3.7
Weeks of pregnancy (mean ± SD)	15.4 ± 2.8	15.8 ± 2.7	15.4 ± 2.9
Pregnant before (% yes)	54.2	53.8	54.4
Gestational diabetes mellitus (%)	13.5	19.2	11.4
Smoking behaviour (% yes)	10.3	19.2	7.2
Any alcohol consumption (% yes)	6.3	4.0	7.2
Ethnicity (% white/Caucasian)	75.5	61.5*	81.4*
Household composition (% living with partner)	85.3	76.0	88.2
Education (% ≥ vocational education)	81.6	84.6	80.0
Occupational status (%)			
Home duties	5.2	3.8	5.8
Unemployed/not able to work	17.5	19.2	17.4
Working (fulltime/part-time)/student	77.3	76.9	76.8
Perceptions and attitude^b^ (median (IQR))			
Attitude to current weight	8.0 (6–9)	8.0 (6–10)	7.0 (6–9)
Attitude to physical activity	9.0 (7–10)	10.0 (8–10)*	8.0 (7–10)*
Self-efficacy	21.0 (17–24.)	21.0 (15–24)	21.0 (17–24)
Social support	8.0 (6–10)	7.0 (5–8)*	8.5 (6–10)*
Outcome expectancy	18.0 (15–20)	18.0 (17–20)	17.0 (14–20)
Cambridge Worry Scale (total 13 items) (median (IQR))	19.0 (11–28)	28.0 (20–40)**	15.0 (10–25)**
Cambridge Worry Scale dimensions (median (IQR))			
Social-medical	5.0 (2–10)	8.5 (4–12)*	4.0 (2–9)*
Socio-economic	4.0 (1–8)	7.5 (2–10)*	4.0 (1–6)*
Health	9.0 (5–12)	13.0 (9–15)**	7.0 (4–10)*
Relationships	0.0 (0–3)	4.0 (0–5)**	0.0 (0–1)**

* *P* < 0.05; differences between depressed mood and good well-being

** *P* < 0.001; differences between depressed mood and good well-being

^*a*^Data from WHO-5 were missing for 2 participants

^b^Based on the HAPA model

Physical activity levels are displayed in Table 2. On average, women wore the accelerometer for almost 13 h (773.8 min) per day, and were sedentary 62.3 % of that time. Fifty percent of the women spent fewer than 23 min (inter quartile range 12.3–35.7) in MVPA per day. Women with a good well-being were involved in MVPA approximately 24 min per day, while women with a depressed mood spent about 13 min per day in MVPA (*P* < 0.05). The time spent in MVPA did not differ between women with more pregnancy-related worries compared to women with less worries (*P* = 0.74).

**Table 2:** Physical activity levels for total sample and stratified for the different categories of the WHO-5 well-being index and Cambridge Worry Scale

		Total	WHO-5 well-being index		Cambridge worry scale		
			Depressed mood	Good well-being	Many worries	Some worries	No or few worries
Minutes of wear time per day	mean (SD)	773.8 (91.1)	771.5 (92.6)	772.2 (91.0)	775.9 (92.7)	775.1 (93.2)	759.7 (83.7)
MVPA^a^ minutes per day	median (IQR)	23.1 (12.3–35.7)	13.3* (8.6–30.0)	24.4* (13.1–38.4)	21.3 (10.8–30.3)	24.0 (12.2–40.8)	24.3 (14.1–35.1)
	Proportion of total wear time (%)	3.4 (2.8)	2.4* (2.1)	3.6* (2.8)	2.9 (1.9)	3.6 (3.1)	3.6 (3.0)
Sedentary behaviour^b^ minutes per day	mean (SD)	484.2 (22.3)	506.2 (96.2)	479.0 (81.1)	477.0 (79.7)	495.3 (95.3)	485.8 (86.1)
	Proportion of total wear time (%)	62.3 (9.7)	65.1 (11.2)	61.7 (8.6)	61.5 (10.0)	63.3 (9.6)	63.4 (9.0)

*P < 0.05; differences between depressed mood and good well-being

^a^Moderate-to-vigorous activity (≥1952 counts per minute)

^b^ < 100 counts per minute

Table 3 shows the results of the linear regression analyses for mental health in association with time spent in MVPA (minutes/day) and sedentary behaviour (proportion of total wear time). In the adjusted model, women with good well-being spent 70 % more minutes per day in MVPA compared women with a depressed mood (*P* = 0.04). Adding a random intercept for country did not (significantly) improve the model, and was therefore not included in the models. Adding HAPA factors (self-efficacy, attitude and social support) did not reduce the association between depressed mood and physical activity. In this model (model 2), women with good well-being spent 85 % more time in MVPA compared to women with depressed mood (*P* = 0.03) (Table 3). Pregnancy-related worries were not significantly associated with MVPA in crude or corrected models (Table 3), and neither aspects of mental health were associated with sedentary behaviour (Table 3).

**Table 3:** Linear regression analyses examining the relationship of mental health with moderate-to-vigorous activity (MVPA) (minutes/day) and sedentary behaviour (% of wear time)

Ln(MVPA)	Ratio	95 % CI	*p*-value
*Crude model*			
Good well-being vs depressed mood	1.72	0.99 – 2.99	0.06
Many worries (ref)	1.00		
Some worries	1.27	0.72 – 2.23	0.40
No or few worries	1.18	0.64 – 2.19	0.59
*Model 1*			
Good well-being vs depressed mood	**1.70**	**1.02 – 2.82**	**0.04**
Many worries (ref)	1.00		
Some worries	0.97	0.58 – 1.62	0.89
No or few worries	0.92	0.52 – 1.61	0.77
*Model 2*			
Good well-being vs depressed mood	**1.85**	**1.06 – 3.24**	**0.03**
Many worries (ref)	1.00		
Some worries	0.94	0.55 – 1.61	0.83
No or few worries	1.01	0.57 – 1.80	0.97
Sedentary behaviour	Difference	95 % CI	*p*-value
*Crude model*			
Good well-being vs depressed mood	−16.82	−60.76 – 27.13	0.45
Many worries (ref)	0		
Some worries	8.05	−35.78 – 51.89	0.72
No or few worries	−18.62	−66.52 – 29.27	0.44
*Model 1*			
Good well-being vs depressed mood	−18.94	−63.95 – 26.08	0.41
Many worries (ref)	0		
Some worries	4.88	−40.01 – 49.76	0.83
No or few worries	−16.17	−64.86 – 32.51	0.51
*Model 2*			
Good well-being vs depressed mood	−36.24	−85.07 – 12.60	0.14
Many worries (ref)	0		
Some worries	1.70	−43.34 – 46.74	0.94
No or few worries	−13.43	−62.25 – 35.39	0.59

Model 1: Corrected for BMI, smoking behaviour, and alcohol consumption

Model 2: Corrected for BMI, smoking behaviour, alcohol consumption, attitude weight, attitude physical activity, self-efficacy, social support and outcome expectancies

Bold font indicates statistically significant associations (p< 0.05).

## Discussion

To the best of our knowledge, we are the first to have studied the associations between emotional well-being and pregnancy-related worries on the one hand, and physical activity, and sedentary behavior on the other, in obese pregnant women across different European countries. Results show that in obese pregnant women, 27 % had depressed mood and these women were significantly less involved in MVPA compared to women with a good well-being. Interestingly, pregnancy-related worries did not significantly impact MVPA levels.

The fact that we measured physical activity with an accelerometer that provides objective information about intensity and duration of different physical activity in daily life [47] is a strength of the study. Previous studies regarding the association between mental health and MVPA used physical activity questionnaires or diaries that have a low correlation with objective physical activity measurement [7, 8]. In addition, these studies were mostly performed in a normal-weighted pregnant population, while our study only included women with a higher BMI who may have worse mental health and lower MVPA levels [4, 5, 24]. Despite these methodological differences, our findings corroborate earlier observations suggesting that a depressed mood is a barrier to be active or to become more active [5, 31].

In our study we operationalized mental health along two dimensions: emotional well-being (depressed mood) and pregnancy-related worries, using validated questionnaires. In our population, 27.1 % of the women had a depressed mood, indicating likely depression, which is somewhat higher than found in Sweden by Claesson and colleagues, who reported around 18 % depression in obese women who were 15 weeks pregnant [48]. In a recent meta-analyses, the median prevalence of depressive symptoms in pregnant women who were obese was 33 %, compared to 22.6 % in normal-weight pregnant women [25]. Differences in depression prevalence may be attributed to case-mix or instruments used. For instance, in the study of Claesson *et al.* [48], fewer women smoked, more women lived together with a partner, and depressive symptoms were measured with the Edinburgh Postnatal Depression Scale [49].

The CWS, used to assess pregnancy-related worries, was found to be highly correlated with trait anxiety, and especially the cognitive element [28]. Our study shows no association with MVPA, suggesting that ‘anxious thoughts’ are not *per se* (linear) related to physical activity. This is in line with earlier findings [2, 8]. Anxious pregnant women may be more restless and nervous, which may lead to increased activity levels. On the other hand, women who have high concerns about the baby’s health and miscarriages may limit their physical activity purposively [31]. The relationship may indeed be curvilinear. Exploring the association between (extreme high and low) anxiety and physical activity behavior in pregnancy may be a fruitful area for future research.

Correction for possible confounders in analyses with MVPA and mental health led in the first step only to correction for lifestyle factors (BMI smoking behaviour, alcohol consumption). Remarkably, no interference with socio-economic factors was found, while previous studies have identified these as important predictors of both mental health and exercise behaviour [7, 17, 50, 51]. A possible explanation for our different result may be that our sample was relatively well-educated and employed (81.6 % with at least vocational education and 77.3 % had work) and were interested in participating in a lifestyle intervention study.

In a second step, all HAPA factors were added to the models. Although these factors led to some change in the effect estimate, this did not reduce the strength of the associations. The measured perceptions and attitudes, although interesting in their own right, did not explain the association between mental health and physical activity.

In our analyses we did not find a clustering effect between the European countries in the association. This may imply an Europe-wide homogeneity of the associations in obese pregnant women with different cultural background. An explanation for this finding is that included countries, albeit with different cultures, were all Western societies with highly similar attitudes towards weight and physical activity in pregnancy [13, 26]. Future studies in a more culturally heterogeneous sample may shed light on the issue of perceptions and stigmas around weight gain, physical activity and obesity in pregnancy.

Some limitations of our study should be acknowledged. We included women who consented to take part in the DALI study and are therefore likely to be motivated to change their physical activity behaviour compared to women who declined to participate. Selection bias may have led to an underestimation of the studied associations.

The use of accelerometer in (obese) pregnant women has been subject of discussion [52, 53]. Due to higher waist circumference and the abdominal shape of obese pregnant women, the accelerometer may change position and measurement may be less accurate. Nevertheless, measuring MVPA by accelerometry may be the best choice in an intervention that promotes physical activity in obese pregnant women [52]. In addition, the Freedson cut-off points were used to determine different activity levels. It is arguable if these cut-off points are adequate in our obese pregnant women, because no golden standard is available and optimal cut-off points may vary across populations [7, 52]. Another concern in our study was that there were some women (*n* = 34) with not enough valid data or software problems. Additional limitations are that no data on prepregnancy physical activity or mental health were available, no data on pregnancy-related symptoms such as nausea, and that prepregnancy BMI was based on self-reported data. Furthermore, the study had a relative small sample size, and was imbalanced, in addition, with only 26 women with depressed mood.

Of course we need to be cautious in interpreting our data, as they are cross-sectional and we cannot exclude reversed causality. Low levels of physical activity may induce or maintain depressed mood and increase pregnancy-related worries. Future studies should aim to assess the association between mental health status and MVPA longitudinally and determine the causal relationship. Because worries and emotional well-being may vary over time, and physical activity levels may decrease with progressing pregnancy, associations may also change.

## Conclusion

Although women with good well-being spent significantly more minutes in MVPA than those who with a depressed mood, still in our sample only 29.6 % of the women complied with the recommendations of MVPA for least 30 min per day. There is ample room for improvement in the total population of pregnant women who are obese. This is exactly the purpose of the DALI study, where pregnant women are offered lifestyle counselling based on principles of motivational interviewing [32].

However, for pregnant women who are obese and have a depressed mood there is a stronger need for improving physical activity behaviour. The combined risk of having poor mental health, obesity and low physical activity levels makes this group extra vulnerable for pregnancy complications. The short WHO-5 might be a suitable instrument for screening in clinical practice. Whether a lifestyle intervention is similarly effective in women with good well-being and those with a depressed mood remains to be seen. Possibly, poor mental health is a barrier for improving lifestyle. Therefore, longitudinal analyses of the association of mental health and changes in physical activity after lifestyle intervention are warranted. However, based on our findings there is a need for focused efforts for the promotion of physical activity among obese pregnant women with at-risk levels of depressive mood.
